# Dalfampridine in the treatment of multiple sclerosis: a meta-analysis of randomised controlled trials

**DOI:** 10.1186/s13023-021-01694-8

**Published:** 2021-02-15

**Authors:** Enyao Zhang, Xin Tian, Ruoming Li, Chaoyang Chen, Min Li, Lingyun Ma, Ran Wei, Ying Zhou, Yimin Cui

**Affiliations:** 1 Department of Pharmacy, Peking University First Hospital, 6 Dahongluochang Street, Xicheng District, Beijing, 100034 China; 2 Department of Pharmacy Administration and Clinical Pharmacy, School of Pharmaceutical Science, Peking University, Beijing, China

**Keywords:** Multiple sclerosis, Dalfampridine, Meta-analysis

## Abstract

**Background:**

Multiple sclerosis (MS) is a chronic illness involving the central nervous system (CNS) that is characterised by inflammation, demyelination, and degenerative changes. Dalfampridine is one of the available treatments for MS symptoms and comorbidities. This meta-analysis aimed to assess the safety and benefits of dalfampridine versus placebo in MS by summarising data deriving from previously published clinical randomised controlled studies (RCTs).

**Results:**

A total of 9 RCTs were included in this meta-analysis, involving 1691 participants. There were significant differences between dalfampridine and placebo in terms of decreased 12-item Multiple Sclerosis Walking Scale score (weighted mean difference [WMD] =  − 3.68, 95% confidence interval [CI] [− 5.55, − 1.80], *p* = 0.0001), improved response to the timed 25-foot walk test (relative risk [RR] = 2.57, 95% CI [1.04, 6.33], *p* = 0.04), increased 6-min walk test (WMD = 18.40, 95% CI [1.30, 35.51], *p* = 0.03), increased 9-Hole Peg Test score (WMD = 1.33, 95% CI [0.60, 2.05], *p* = 0.0004), and increased Symbol Digit Modalities Test score (WMD = 4.47, 95% CI [3.91, 5.02], *p* < 0.00001). Significant differences in the incidence of side effects were also observed (RR = 1.12, 95% CI [1.04, 1.21], *p* = 0.002).

**Conclusion:**

Dalfampridine exerts positive effects on walking ability, finger dexterity, and cognitive function. Treatment should be administered under the guidance of a physician or pharmacist given the higher incidence of adverse events.

## Background

Multiple sclerosis (MS) is a chronic illness involving the central nervous system (CNS) that is characterised by inflammation, demyelination, and degenerative changes. Most people who are diagnosed with MS are between the ages of 20 and 40 years old and the number of women is 2- or 3-times higher than the number of men [1]. With a prevalence of 50–300 per 100,000 people, MS affects about 2.3 million people globally [1]. As one of the most frequent causes of non-traumatic disability in young people, it represents a significant burden in terms of impact on the quality of life, societal costs, and personal expenses [2, 3]. MS can cause a wide range of symptoms that can vary widely from person to person and is accompanied by periodic changes in severity. Common symptoms include weakness in the limbs, problems with gait and movement, sensory disturbances, fatigue, visual difficulties, cognitive deficits, and increased neuropathic discrepancies [4].

Currently, there is no curative treatment available for MS. Its treatment only includes disease-modifying therapies, which tend to be MS-specific, and symptomatic therapies, which are often used in different disease areas to treat symptoms resulting from neurological dysfunction [5]. Although disease-modifying treatments (DMTs) are effective in reducing the risk of relapses and potentially the progression of disability, they cannot address the poor quality of life, which largely contributes to non-persistence and discontinuation rates for DMT treatment [6, 7]. Given this, it is crucial to adopt specific treatment for MS symptoms.

Dalfampridine is an extended-release form of 4-aminopyridine (4-AP), a broad-spectrum lipophilic potassium channel blocker that binds preferentially to the open state of the potassium channel in the CNS. Its pharmacological targets are the potassium channels exposed in MS patients; therefore, it can restore conduction in focally demyelinated axons. 4-AP also increases calcium (Ca^2+^) influx at presynaptic terminals, thereby enhancing neuroneuronal or neuromuscular transmission in normally myelinated neurons. These pharmacological properties have prompted extensive investigation of its therapeutic potential for symptom management in disorders of neuromuscular transmission and in demyelinating diseases such as spasticity in hereditary spastic paraparesis [8]. In 2010, the United States Food and Drug Administration (FDA) approved dalfampridine as treatment to improve walking, and many clinical trials have shown its positive efficacy [9]. Dalfampridine can also be used as medication for other MS symptoms and comorbidities such as upper limb impairment and cognitive fatigue. Since it does not interact with DMTs, dalfampridine can be seen as a complement to DMTs.

The effects of dalfampridine in difficulty walking are well-documented; however, little is known about its other functional effects including hand use and mental functions. Since 2014, evidence-based clinical studies have focused solely on walking disability or lack quantitative synthesis [10, 11]. Furthermore, new clinical trials published after 2019 can be added to offer more accurate conclusions. The purpose of this meta-analysis was to evaluate the overall effects of dalfampridine derived from published studies.

## Materials and methods

### Search strategies

Electronic searches of the following databases were conducted: PubMed, Embase, the Cochrane Central Register of Controlled Trials and ClinicalTrials.gov (all available years to August/2020). The keywords of the search strategies were ‘4-AP’, ‘4-aminopyridine’, ‘fampridine’, ‘dalfampridine’, ‘Fampyra’, ‘Multiple Sclerosis’, ‘Sclerosis’, ‘Multiple’, ‘Disseminated’, and ‘Disseminated Sclerosis’. The authors together developed the full search strategy combining medical subject headings (MeSH)/Emtree terms (in Embase) and free-text terms in order to search all possible articles, and the final strategy was adapted to the database search (Additional file 1).

### Selection of studies

Two authors independently screened titles and abstracts of the citations retrieved from the literature search for inclusion/exclusion of the studies and obtained the full text of potentially relevant studies for further assessment. A third reviewer resolved any disagreements arising between them. The authors excluded all irrelevant records and noted details of studies and reasons for their exclusion.

Studies meeting the following criteria were included: (1) randomised, double blind, placebo-controlled trials; (2) studies of patients with MS diagnosis according to the McDonald's criteria [12, 13], over 18 years of age, and with any score on the Expanded Disability Status Scale (EDSS) [14]; (3) studies including all subgroups of MS (relapsing–remitting, secondary-progressive, primary-progressive, and progressive-relapsing), regardless of sex, degree of disability, and disease duration of the individual; and (4) studies reporting at least one of the following outcomes: the 12-item Multiple Sclerosis Walking Scale (MSWS-12); responders to treatment based on ≥ 20% improvement in walking speed (in feet per second), as measured by the timed 25-foot walk (T25FW); the 6-min walk (6-MW), defined as the number of meters a patient can walk in 6 min; the 9-Hole Peg Test (9-HPT), which is used to measure finger dexterity; the Symbol Digit Modalities Test (SDMT); and the incidence of any adverse events (AEs). We included all trials in which dalfampridine, with no restrictions on the dose, could be compared with placebo, with no restrictions on placebo type. Concomitant DMTs were allowed if they were used equally in all intervention groups in the trial. Studies were excluded if one of the following conditions was met: the language of the publication was not English; the study design was an observational, retrospective study, a cross-over study, repeated published research, a conference summary, a case analysis, a literature review, comment or involved animal experiments; the study disease was not MS; the intervention of original research was not dalfampridine or other forms of dalfampridine; the study control was not a placebo; and the research data were missing too much or were not available.

To avoid the impact of duplicate data or samples on estimate efficacy and safety, we excluded articles with shared authorship.

### Data extraction and quality assessment

When the literature was screened, review authors independently extracted the related data using a data extraction form structured to include basic research information, baseline characteristics of the study subject, results data, and quality evaluation information. We contacted the author if the data were not fully reported, and measured data from the graphs using digital ruler software if the data were only expressed graphically. Any dispute was discussed and resolved by a third reviewer.

Two authors independently assessed the risk of bias of included studies using the Cochrane risk-of-bias tool. The tool consists of the following items: sequence generation, allocation concealment, blinding of participants and personnel, blinding of outcome assessment, incomplete outcome data, selective outcome reporting, and other bias. A third reviewer was responsible for resolving any discrepancies if they arose.

### Data synthesis and statistical analysis

Data analysis was performed using Cochrane Systematic review software Review Manager (Rev-Man; Version 5.3). Continuous variables were presented as weighted mean differences (WMDs), which are also known as mean differences (MDs), with 95% confidence intervals (CIs), and dichotomous variables were presented as relative risks (RRs) with 95% CIs. As the units of measurement for continuous outcomes of interest were the same across studies, we used only WMDs for continuous variables instead of standardised mean differences (SMDs). The heterogeneity between the included studies was analysed by the *I*^2^ test. Substantial heterogeneity was defined as *I*^2^ > 50%. Random-effects models were applied with the presence of heterogeneity. Otherwise, the fixed-effect model was used. Since we included less than 10 studies for data synthesis of each outcome, we were not permitted to use a funnel plot to explore possible publication bias. In the case where there was evidence of trials results heterogeneity, we planned to perform a sensitivity analysis to determine the effect of excluding trials with a high risk of bias.

## Results

### Search results

**Fig. 1:**
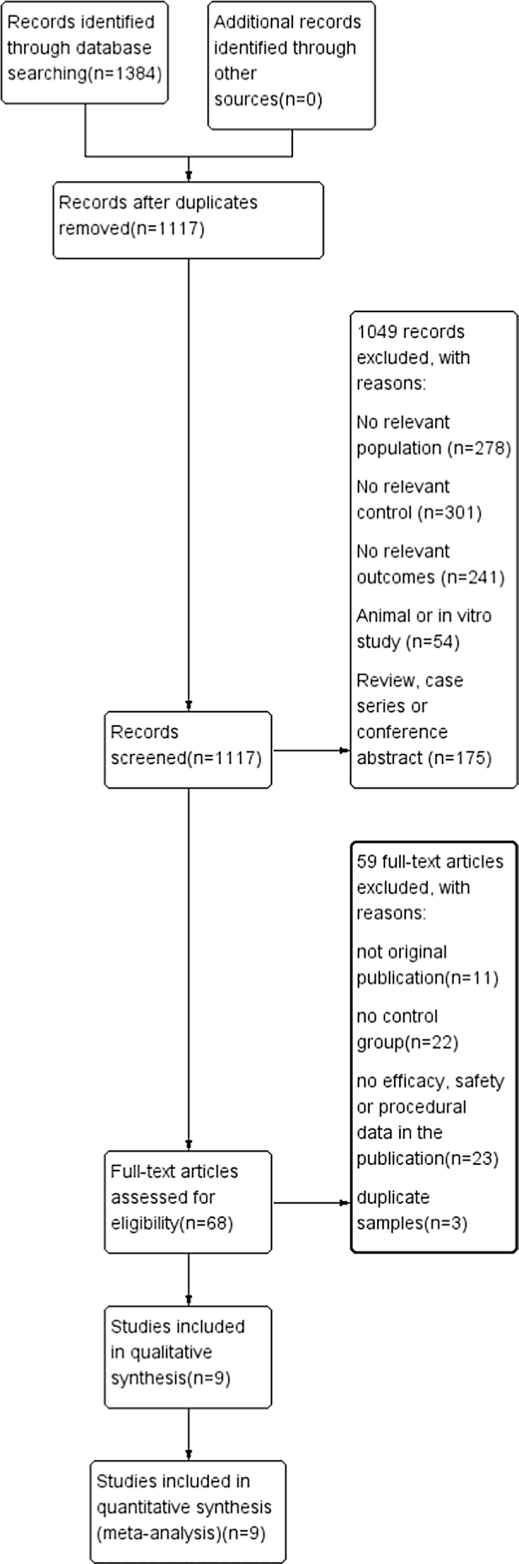
Flow chart the study selection process

After the initial search, a total of 1384 studies related to dalfampridine from 4 databases were retrieved. We reviewed the full text of 68 reports after reading the title and abstract, of which 59 reports were excluded based on the exclusion criteria. Therefore, 9 randomised controlled trials (RCTs) were finally selected for this meta-analysis. The results of the search and selection process are shown in Fig. 1.

### Study characteristics

The sample size ranged from 21 to 633 and average age ranged from 39.5 to 54.0 years. In these studies, the intervention groups received dalfampridine and the control groups received placebo. The dose of dalfampridine was between 5 and 10 mg taken orally twice daily. Duration of treatment ranged from 4 to 24 weeks. The basic characteristics of included trials in this meta-analysis were summarised in Table 1.

**Table 1:** Basic characteristics of the included studies

Study	Research type	Intervention	DMT use	Dose	Number (I/C)	Women (I/C)	Age (I/C)	EDSS (I/C)	Treatment course	Outcome indicator
Jeremy Hobart2019 [15]	RCT	Dalfampridine/Placebo	Y	10 mg twice daily	315/318	186/180	49.0/48.8	5.49/5.48	24 weeks	①⑥
Francois Jacques2018 [16]	RCT	Dalfampridine/Placebo	Y	10 mg twice daily	21/20	11/10	54.05/50.30	4.62/4.82	14 weeks	②③
Raymond Hupperts2016 [17]	RCT	Dalfampridine/Placebo	Y	10 mg twice daily	68/64	38/33	49.8/49.8	5.6/5.9	24 weeks	①⑥
H.B Jensen2016 [18]	RCT	Dalfampridine/Placebo	NA	10 mg twice daily	16/19	8/12	50.8/48.4	5.8/5.5	26–28 days	④⑤
Robert Yapundich2015 [19]	RCT	Dalfampridine/Placebo	NA	5, 10 mg twice daily	287/143	200/200	52.8/52.2	4.8/4.8	4 weeks	①②③⑥
A.D. Goodman2010 [20]	RCT	Dalfampridine/Placebo	Y	10 mg twice daily	120/119	88/74	51.8/51.7	5.8/5.6	9 weeks	①②⑥
C. Arreola-Mora2019 [21]	RCT	Dalfampridine/Placebo	NA	10 mg twice daily	11/10	7/6	39.5/39.3	4.7/4.3	20 weeks	⑤⑥
Laura De Giglio2019 [22]	RCT	Dalfampridine/Placebo	NA	10 mg twice daily	80/40	50/24	49.3/46.7	4/4.5	12 weeks	④⑤⑥
Simpson Marion2020 [23]	RCT	Dalfampridine/Placebo	NA	10 mg twice daily	20/20	12/12	53.5/51.5	NA	8 weeks	④

①MSWS-12②Responders to T25FW③6-MW④9-HPT⑤SDMT⑥Any adverse event rate; *I* intervention (dalfampridine), *C* control (placebo), *Y* yes, *N* no, *NA* not available

### Risk of bias

**Fig. 2:**
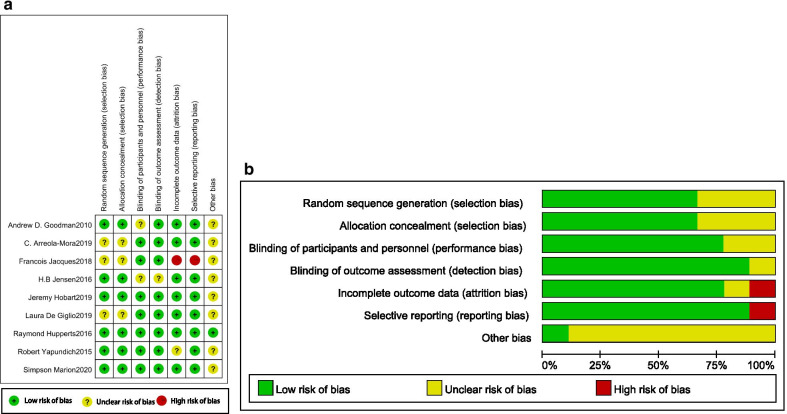
**a** Risk of bias summary: review authors' judgements about each risk of bias item for each included study. **b** Risk of bias graph: review authors' judgements about each risk of bias item presented as percentages across all included studies

Seven aspects of the RCTs related to the risk of bias were assessed, following the instructions in the Cochrane Handbook for Systematic Reviews of Interventions. Figure 2a, b summarise the characteristics of the risk of bias of included studies. All 9 studies mentioned randomness, but only 6 studies described the methods of random number generation in detail. The majority of studies clearly reported the methods of allocation concealment and blinding of outcome assessors. Only 1 study did not mention the results completely, showing great selective reporting bias. In summary, we judged the overall risk of bias of included trials to be acceptable.

### Meta-analysis results

#### MSWS-12

**Fig. 3:**
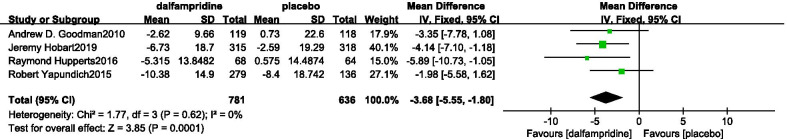
Forest plot of the MSWS-12 score change from baseline with dalfampridine and placebo

A total of 4 trials [15–18] reported MSWS-12 score changes of dalfampridine versus placebo. Yapundich et al. [17] used different doses of dalfampridine in comparison with placebo. Since no significant differences were found across different doses, we included them as a whole experimental group. There were in total of 781 patients in the dalfampridine test group and 636 patients in the placebo control group. A fixed-effects model was adopted because no significant heterogeneity was identified (χ^2^ = 1.77, *df* = 3, *p* = 0.62, *I*^2^ = 0%) (Fig. 3), and there were significant differences between the dalfampridine test group and the placebo control group in terms of decreased MSWS-12 score (WMD =  − 3.68, 95% CI [− 5.55, − 1.80], *p* = 0.0001) (Fig. 3).

#### Response to T25FW

**Fig. 4:**
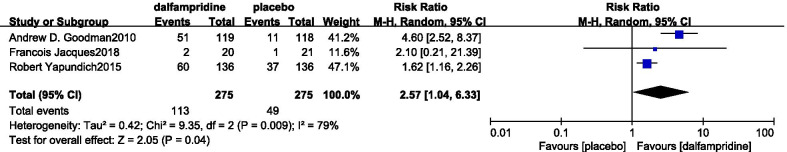
Forest plot of response to T25FW with dalfampridine and placebo

Three trials [17, 18, 20] reported data for the response to T25FW. There was great heterogeneity between studies (χ^2^ = 9.35, *df* = 2, *p* = 0.009, *I*^2^ = 79%) (Fig. 4), so we used the random-effects model to pool the data. With significant differences among groups of different doses in Yapundich et al. [17], we included only data of participants given 10 mg twice daily. There were in total of 275 patients in the dalfampridine test group and 275 patients in the placebo control group. The overall estimate indicated that the pooled RR was 2.57 (95% CI [1.04, 6.33], *p* = 0.04) (Fig. 4). Thus, dalfampridine significantly improved the clinical response to T25FW compared with placebo.

#### 6-MW

**Fig. 5:**
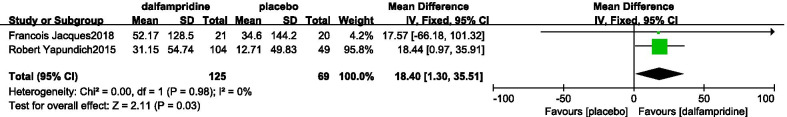
Forest plot of the 6-MW change from baseline with dalfampridine and placebo

Two trials [17, 20] showed the 6-MW changes of dalfampridine versus placebo. Since no significant difference was found among groups of different doses in Yapundich et al. [17], we included them as a whole experimental group. There were in total 125 patients in the dalfampridine test group and 69 patients in the placebo control group. Meta-analysis results using the fixed-effects model show that the average change in 6-MW in the dalfampridine test group was significantly higher than the placebo control group (WMD = 18.40, 95% CI [1.30, 35.51], *p* = 0.03) (Fig. 5).

#### The 9-Hole Peg Test

**Fig. 6:**
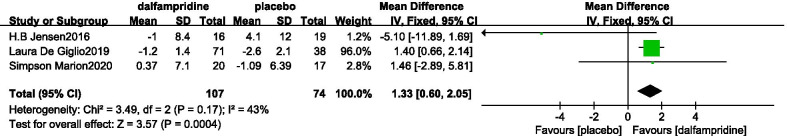
Forest plot of the 9-HPT change from baseline with dalfampridine and placebo

Three trials [19, 21, 22] reported results for changes in the 9-HPT following treatment with dalfampridine and placebo. There were in total 107 patients in the dalfampridine test group and 74 patients in the placebo control group. A fixed-effects model was adopted. In summary, significant differences in the change of the 9-HPT test score was found between the 2 groups (WMD = 1.33, 95% CI [0.60, 2.05], *p* = 0.0004) (Fig. 6).

#### Symbol Digit Modalities Test

**Fig. 7:**
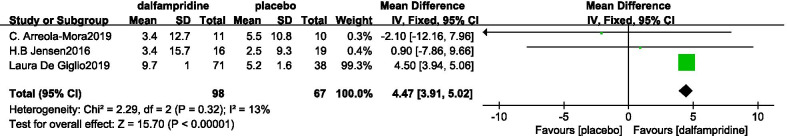
Forest plot of the SDMT score change from baseline with dalfampridine and placebo

Three trials [19, 21, 23] compared changes in the SDMT score in the dalfampridine test group and the placebo control group. In total, 98 patients were treated with dalfampridine and 67 with placebo. A fixed-effects model was used and the present meta-analysis revealed that there was significant difference between 2 groups in terms of the change in SDMT score (WMD = 4.47, 95% CI [3.91, 5.02], *p* < 0.00001) (Fig. 7).

#### Incidence of adverse events

**Fig. 8:**
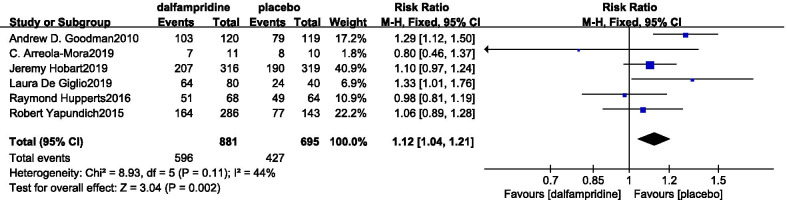
Forest plot of any adverse events incidence with dalfampridine and placebo

This meta-analysis included 6 trials [15–18, 21, 23] on dalfampridine versus placebo with any AEs, including urinary tract infection, fall, insomnia, headache, asthenia, dizziness, nausea, back pain, balance disorder, upper respiratory tract infection, arthralgia, cough, nasopharyngitis, paraesthesia, spasticity, mood alteration, gastric pain, vertigo, hand tremor, blurring of vision, pain in the eye, pain in the extremity, calf cramps, oedema, sciatica, and influenza. The AEs of treatment group were 596 and the total incidence rate was 67.6%, while in the control group was the number of AEs was 427 with a total incidence rate of 61.4%. Adopting a fixed-effects model, significant differences of the incidence of side effects were observed (RR = 1.11, 95% CI [1.05, 1.18], *p* = 0.0006) (Fig. 8).

### Publication bias and sensitivity analysis

We did not perform a funnel plot because less than ten trials were included in each meta-analysis. To evaluate the effect of each study of a high risk of bias on the pooled results with great heterogeneity, we conducted sensitivity analysis by omitting each study, one at a time. Great heterogeneity in response to T25FW may come from the difference in basic characteristics of participants such as race, sex, age, EDSS score, and concomitant DMT use. A study by Yapundich et al. [17] was found to be the contributor of heterogeneity as *I*^2^ reduced from 79 to 0% after excluding this study. A possible explanation might be that the patient population included in this study was limited to only American patients. Furthermore, the baseline in walking ability of the study by Yapundich et al. [17] was not limited, but it could also be explained by the variation in the study aims, study designs, and the definitions of outcomes. However, the sensitivity analysis showed that the RRs were 2.57 (95% CI 1.04, 6.33) and 4.38 (95% CI 2.45, 7.82) before and after the removal of that study, respectively, indicating high stability of the results. The results showed that the stability of results had no significant changes, which validated the rationality and reliability of our analysis.

## Discussion

This meta-analysis included 9 RCTs examining the effect of dalfampridine on functioning in patients with MS. The overall population included 1691 participants, and the quality of the studies was good.

The pooled data of MSWS-12 score changes suggested that dalfampridine was associated with slight improvement in walking ability in MS patients. However, according to a research by Hobart et al. [24], the minimal clinically important difference for the MSWS-12 was estimated to be 3.9 to 5.1 points. After WMDs were compared between treatment groups, and although the results were statistically significant, there is no equivalent impact on clinical meaningfulness. Although the MSWS-12 is a validated patient-reported outcome measure for assessing the extent to which MS impacts an individual’s walking ability [25], few studies have explored the minimal clinically important differences for the tool. As some included studies [15, 16] reported significant clinical differences in MSWS-12 for patients receiving dalfampridine versus placebo, future research in this area is required to explore the definite effect of dalfampridine on walking disability and determine the minimal clinically important differences for MSWS-12. T25FW and 6 MW are measures of short- and long-distance ambulatory function, respectively [26]. In our meta-analysis, dalfampridine could significantly improve the response to T25FW and 6 MW suggesting dalfampridine has a positive effect on both short distance and long distance. Despite the varying degrees of improvement, it is meaningful for MS patients to be given dalfampridine as a supplementary medicine given its more convenient formulation. A prior meta-analysis has evaluated the effect of dalfampridine on mobility disability, but we itemised different aspects of mobility disability and used different outcomes [10].

Furthermore, walking disability is one of the most prevalent symptoms in MS patients, experienced by over 50% of individuals with MS [27]. It has a significant adverse impact on health-related quality of life and employment [28, 29]. Considering the broad impact on daily life, and on personal and social economic burden, it is necessary to manage this symptom properly. However, over 60% of outpatients with walking difficulty are overlooked by their physicians [30]. It is also important to note that MS patients were reluctant to ask help for walking disability from their doctors [31]. All these factors led to the current statement of poor management in walking disability and insufficient studies in this area. Our study not only provided a feasible approach to mitigate walking difficulty, but also aroused public attention for improved care in this issue.

We used 9-HPT to measure finger dexterity in patients and our results indicated that significant change was found in the 9-HPT test score in patients treated with dalfampridine, which meant dalfampridine was beneficial to hand use. A large number of individuals with MS experience limb weakness and their manual abilities are inevitably affected. This deficit may be associated with tremor, coordination deficits, muscle weakness, and deconditioning [32, 33]. The daily life of MS patients is severely interrupted as they cannot perform basic activities such as dressing, bathing, and selfcare independently. Because of the loss of independence, patients participate in fewer social activities and have lower quality of life. Furthermore, individuals with difficulty in hand use are more likely to become unemployed, which constitutes a heavy economic burden [33]. There is a correlation between increasing 9-HPT scores and increasing annualised direct costs associated with MS, including fees for doctor's visits, medications, necessary changes to cars or homes, and ultimately long-term care [34]. Therefore, physicians need to focus more on improving impaired arm function in patients with MS. Previous studies [35, 36] have focused on physical therapy, which had limited effects and could not be accomplished at home. Dalfampridine may provide a new potential as an oral drug to improve 9-HPT and manage this symptom. However, the statistically significant difference in 9-HPT found in our study did not indicate a clinically meaningful difference. A study by Hervault et al. [37] revealed that the minimal detectable change in 9-HPT time was 4.38 s, which meant our results would be difficult to apply in clinical practice. Some open-label studies [38–40] have reported small but statistically significant decreases in 9-HPT time, and in no case was there an associated clinical improvement for this measure. Our study produced similar results, although some limitations of our study, including the small sample size and heterogeneity of the patient group with regard to the type of upper limb dysfunction, may have interfered with the interpretation of those results. As the dalfampridine test group clearly demonstrated prolonged 9-HPT times, future research in this area might include stratification according to the type of upper limb dysfunction. The effects of drug treatment combined with physical therapy interventions could also be explored.

To test the effects of dalfampridine on cognitive impairment, we compared changes in the SDMT score in the dalfampridine test group and in the placebo control group and found that dalfampridine improved performance in cognitive processes. The prevalence of cognitive changes in MS patients ranged from 33 to 65%, across all MS phenotypes [41–45]. As for other symptoms of MS, cognitive impairment may severely impact on activities of daily life, including work, driving, or management of business affairs. In addition, cognitive deficits are associated with poor adherence to treatment [46]. Thus, it is essential to harmonise the treatment of cognitive function into a cohesive treatment plan. While some studies have concentrated on the relationship between exercise and cognitive rehabilitation, some clinical trials have shown limited benefits of treatments including amantadine, dalfampridine, l-amphetamine, lisdexamfetamine dimesylate, memantine, rivastigmine, and donepezil [47–54]. It is noteworthy that dalfampridine has a relatively better safety profile compared with other medicines [47, 49–54]; furthermore, our meta-analysis confirmed its efficacy, and supports its use as a viable approach to manage cognitive decline in patients.

Spasticity is one of the most important factors contributing to changes in speed and gait quality, which means research investigating the effects of dalfampridine on spastic symptoms in MS patients is meaningful for clinical use. However, we did not report any outcomes concerning spasticity, because there have been no RCTs evaluating the effects of dalfampridine on spasticity of patients with MS. Future studies may fill the knowledge gap in this field.

Concerning the incidence of the AEs, our meta-analysis agreed with conclusions of previous reviews [10, 11]. As dalfampridine can activate the excitatory state of neurons and amplify synaptic transmission throughout the brain and spinal cord, the incidence of AEs of patients receiving dalfampridine was slightly higher than that in the placebo control group. Dalfampridine is known to be substantially excreted by the kidneys and the risk of adverse reactions, including seizures and anaphylaxis, is greater with increasing exposure. According to the 5-year post-marketing data in the United States [55], among the 107,000 patients treated with dalfampridine, 23.9% (25,526) of patients reported at least one AE. Of these patients, 75% were female, and the mean age was 55.3 years; while the proportion of patients aged ≥ 65 years was 19%. The most commonly reported AEs included dizziness (3.7%), insomnia (3.2%), balance disorder (3%), falls (2.4%), headache (2.4%), nausea (2.1%), and urinary tract infection symptoms (2%). Thus, it is recommended that patients younger than 18 and over 65, with impaired renal function or lactating women need to follow doctors’ recommendations and be monitored for any adverse reactions [56].

Despite the good efficacy and safety of dalfampridine, treatment has been mainly limited to patients in western countries, and the drug is rarely included as a potential therapeutic option for the symptomatic treatment of MS in other countries or regions such as Latin America and Asia. We presume there are three main reasons for this difference. First, developing countries may allocate relatively limited financial resources to healthcare and thus may be less interested in advances in the treatment of MS which is classified as a rare disease; thus, they may be unaware of trends in comprehensive care for MS and ignore the significance of symptomatic treatment. Second, the average price of a 10-mg dalfampridine tablet is $21.12, which means a 1-month treatment supply would cost $1267.20 [57]. This entails a significant economic burden on individuals living in developing countries and may force them to abandon symptomatic treatment. Third, the currently available findings on dalfampridine are mainly limited to the Caucasian population, and the effects may not be generalisable to other ethnic populations, which leads to reduced applicability. Future study is needed to expand accessibility to medicines in order to maximise availability for safe and rational use for symptomatic treatment of MS worldwide.

Overall, MS has many disabling symptoms with deleterious consequences on employment, social functioning, and quality of life, which range from walking disabilities to cognitive impairment [58]. Although dalfampridine was first approved to treat disability in movement, some clinical trials [19, 21–23] have found it exerts effects on other bodily functions, which have attracted wider interest. In this study, we used meta-analysis to obtain a valid conclusion of the effects on dalfampridine on different symptoms. To the best of our knowledge, this is the first meta-analysis providing comprehensive insight into the global effects of dalfampridine. Furthermore, we reviewed recent studies into the efficacy and safety of dalfampridine and included only high-quality RCTs and provided high-quality evidence.

However, we should acknowledge this meta-analysis has some limitations. First, confounding bias might have caused varying degrees in the quality of all the included trials and heterogeneity existed among studies. Second, we included fewer than 10 studies in each meta-analysis, and publication bias could not be ignored. Third, some required data for outcomes were missing. We were unable to contact authors to obtain supplementary data and resorted to the use of software to compute effect sizes (with CIs) on outcomes of interest. Few authors responded to our request for additional data, which resulted in a reporting bias across studies. Dose inconsistencies for different studies, complex disease subtypes, changing outcomes of different studies also obstructed our data pooling. Furthermore, we excluded studies in other languages, which may have resulted in missing some important data.

## Conclusions

In conclusion, this meta-analysis showed that dalfampridine has a positive effect on perceived and objectively measured walking capacity, hand use, and mental functions. With a slightly higher incidence of AEs, dalfampridine requires administration under the guidance of a physician or pharmacist. Additional high-quality RCTs trials are needed to verify the results, and equally, long-term studies to evaluate the safety of dalfampridine more completely are needed.

## Supplementary Information

**Additional file 1. **Additional methods: search algorithms.

## Data Availability

The data set supporting the results of this article are included within the article and other supplements.

## Abbreviations

MS: Multiple sclerosis
CNS: Central nervous system
DMTs: Disease-modifying treatments
4-AP: 4-Aminopyridine
EDSS: Expanded Disability Status Scale
MSWS-12: The 12-item Multiple Sclerosis Walking Scale
T25FW: Timed 25-foot walk
6-MW: 6-Min walk
9-HPT: 9-Hole Peg Test
SDMT: Symbol Digit Modalities Test
AEs: Adverse events
