# Supplementary material for: Dalfampridine in the treatment of multiple sclerosis: a meta-analysis of randomised controlled trials
Source: Orphanet J Rare Dis. 2021 Feb 15;16:87. doi: 10.1186/s13023-021-01694-8 (PMC7885571; doi:10.1186/s13023-021-01694-8)
Supplement: Supplementary file 1 — Additional file 1. Additional methods: search algorithms. [file 13023_2021_1694_MOESM1_ESM.docx]

**Supplementary appendix**

Search strategy employed in August 2020 for PubMed

| Database | Step | Search algorithm | Items found |
| --- | --- | --- | --- |
| PubMed | #1  #2  #3  #4  #5  #6  #7  #8  #9  #10  #11  #12  #13 | 4-AP  4-aminopyridine  fampridine  dalfampridine  Fampyra  Multiple Sclerosis  Sclerosis  Multiple  Disseminated  Disseminated Sclerosis  ((((#1) OR (#2)) OR (#3)) OR (#4)) OR (#5)  ((((#6) OR (#7)) OR (#8)) OR (#9)) OR (#10)  (#11) AND (#12) | 7534  6863  6913  6903  11  92453  165010  1447781  155967  92686  7620  1659385  605 |

Query:

("4 aminopyridine"[MeSH Terms] OR "4 aminopyridine"[All Fields] OR "4 ap"[All Fields] OR ("4 aminopyridine"[MeSH Terms] OR "4 aminopyridine"[All Fields] OR "4 aminopyridine"[All Fields]) OR ("4 aminopyridine"[MeSH Terms] OR "4 aminopyridine"[All Fields] OR "fampridine"[All Fields]) OR ("4 aminopyridine"[MeSH Terms] OR "4 aminopyridine"[All Fields] OR "dalfampridine"[All Fields]) OR "Fampyra"[All Fields]) AND ("multiple sclerosis"[MeSH Terms] OR ("multiple"[All Fields] AND "sclerosis"[All Fields]) OR "multiple sclerosis"[All Fields] OR ("sclerosi"[All Fields] OR "sclerosis"[MeSH Terms] OR "sclerosis"[All Fields] OR "scleroses"[All Fields]) OR ("multiple"[All Fields] OR "multiples"[All Fields]) OR ("sclerosi"[All Fields] OR "sclerosis"[MeSH Terms] OR "sclerosis"[All Fields] OR "scleroses"[All Fields]) OR ("disseminate"[All Fields] OR "disseminated"[All Fields] OR "disseminates"[All Fields] OR "disseminating"[All Fields] OR "dissemination"[All Fields] OR "disseminations"[All Fields] OR "disseminator"[All Fields] OR "disseminators"[All Fields]) OR ("multiple sclerosis"[MeSH Terms] OR ("multiple"[All Fields] AND "sclerosis"[All Fields]) OR "multiple sclerosis"[All Fields] OR ("disseminated"[All Fields] AND "sclerosis"[All Fields]) OR "disseminated sclerosis"[All Fields]))
